# Prevalence of MASLD and fibrosis assessed by transient elastography in U.S. adolescents: insights from NHANES 2017-2023

**DOI:** 10.1186/s13098-025-02024-9

**Published:** 2025-12-30

**Authors:** Jialin Wu, Junlong Huang, Shiyu Cao, Yang Lyu, Peiyao Yu, Tiejun Feng, Bonan Chen, Fuda Xie, Ge Zhang, Kangmin Zhuang, Aimin Li, Ka Fai To, Wei Kang

**Affiliations:** 1 Department of Anatomical and Cellular Pathology, State Key Laboratory of Translational Oncology, Sir Y.K. Pao Cancer Center, Prince of Wales Hospital, The Chinese University of Hong Kong, Hong Kong, China; 2 State Key Laboratory of Digestive Disease, Institute of Digestive Disease, Li Ka Shing Institute of Health Science, The Chinese University of Hong Kong, Hong Kong, China; 3 CUHK-Shenzhen Research Institute, Shenzhen, China; 4 Department of Urology, Third Affiliated Hospital of Sun Yat-Sen University, Guangzhou, China; 5 Guanghua School of Stomatology, Sun Yat-sen University, Guangzhou, 510080 Guangdong Province China; 6 School of Chinese Medicine, Law Sau Fai Institute for Advancing Translational Medicine in Bone and Joint Diseases (TMBJ), Hong Kong Baptist University, Hong Kong, China; 7 Guangdong Provincial Key Laboratory of Gastroenterology, Department of Gastroenterology, Nanfang Hospital, Southern Medical University, Guangzhou, China

**Keywords:** Metabolic dysfunction-associated steatotic liver disease, NHANES, Prevalence, Trend

## Abstract

**Background/aims:**

Metabolic dysfunction-associated steatotic liver disease (MASLD) is a common but understudied disease in adolescents. We aimed to estimate the updated prevalence of MASLD and related fibrosis among US adolescents using transient elastography.

**Methods:**

This study analyzed data from the National Health and Nutrition Examination Survey 2017 to March 2020 and August 2021 to August 2023 among adolescents ages 12-19 years. Steatotic liver disease was assessed using the median controlled attenuation parameter (CAP) and fibrosis by median liver stiffness measurement.

**Results:**

A total of 2588 participants were included in the analysis (mean [SD] age, 15.4 [2.3] years; 1366 male participants [52.8%]). The overall age-adjusted prevalence of MASLD was 21.0% (95% CI: 19.1–23.0) using a CAP threshold of ≥ 248 dB/m and 16.1% (95% CI: 14.4–17.8) using ≥ 263 dB/m. The prevalence of MASLD-related fibrosis was 9.0% and 9.7% using CAP thresholds of 248 dB/m and 263 dB/m, respectively. Higher prevalence of MASLD and fibrosis was observed among adolescents with overweight, obesity, and prediabetes. Between the two survey cycles, the age-standardized prevalence of MASLD remained stable, with a non-significant decline observed in the prevalence of fibrosis. Multivariable analysis identified male sex, non-Hispanic Asian ethnicity, increased waist circumference, overweight, and obesity as independent risk factors for MASLD, while waist circumference was the only independent factor associated with MASLD-related fibrosis.

**Conclusions:**

In this cross-sectional study, MASLD and MASLD-related fibrosis were highly prevalent among US adolescents, with significant disparities observed by sex, race, and ethnicity. These findings highlight the substantial burden of MASLD in the adolescent population and underscore the need for continued public health focus.

**Graphical abstract:**

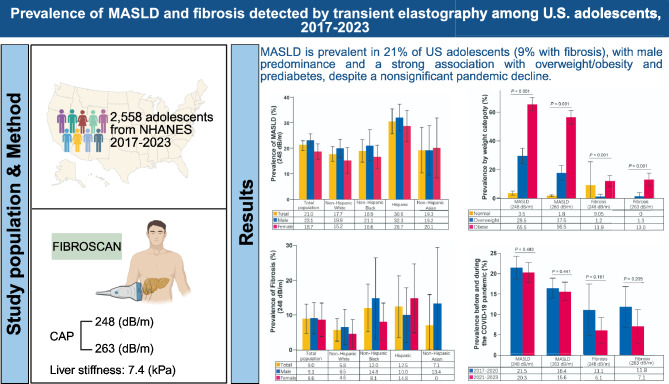

**Supplementary Information:**

The online version contains supplementary material available at 10.1186/s13098-025-02024-9.

## Introduction

Non-alcoholic fatty liver disease (NAFLD) has become a leading cause of chronic liver disease globally and is emerging as the predominant etiology of end-stage liver disease in the coming decades, affecting both adult and pediatric populations [1]. Individuals with NAFLD exhibit a significant association with hepatic fibrosis, cirrhosis, hepatocellular carcinoma (HCC), as well as extrahepatic complications, particularly cardiovascular disease [2–4]. In response to the evolving understanding of its pathophysiology, the 2023 Delphi consensus statement introduced the term "metabolic dysfunction-associated steatotic liver disease" (MASLD) to more accurately reflect this multifaceted disorder characterized by a wide range of clinical phenotypes [5]. This new nomenclature aims to enhance clinical and epidemiological discourse and promote the development of innovative therapeutic approaches. MASLD represents the presence of hepatic steatosis and at least one cardiometabolic risk factor (CMRF), including obesity, diabetes, dyslipidemia and/or hypertension, and in the absence of other identifiable causes of hepatic fat accumulation, such as alcohol consumption or drug intake [5].

In recent years, the prevalence of MASLD among adolescents has significantly increased due to lifestyle changes. Current studies indicate that individuals with characteristics of CMRF exhibit a MASLD prevalence of 80% or higher, accompanied by an elevated mortality rate [6]. Similarly, the prevalence of MASLD is approximately three times greater in children and adolescents who are obese or have type 2 diabetes or prediabetes compared to those of normal weight and glucose tolerance [7–9]. This trend poses a considerable threat to global public health and constitutes a substantial medical burden, underscoring the need for increased focus on the health of adolescents and young adults.

The global COVID-19 pandemic has been found to be significantly associated with an increased risk of MASLD and liver injury [10, 11]. COVID-19 has the capacity to impair energy metabolism both directly and indirectly, contributing to an increase in visceral adipose tissue, hyperglycemia, and overt diabetes [12, 13]. Furthermore, the pandemic has likely altered lifestyle behaviors due to restrictions on outdoor activities, including poor dietary habits and reduced physical activity, further promoting the development of MASLD [14, 15]. However, while research on the pandemic's association with MASLD has advanced in adults, a critical gap remains in understanding its contemporary prevalence among adolescents. This study aims to address this gap by providing updated national estimates of MASLD, diagnosed via the controlled attenuation parameter (CAP) score, and fibrosis prevalence among U.S. adolescents using NHANES data from 2017 to 2023.

## Materials and methods

### Study design and participants

**Fig. 1:**
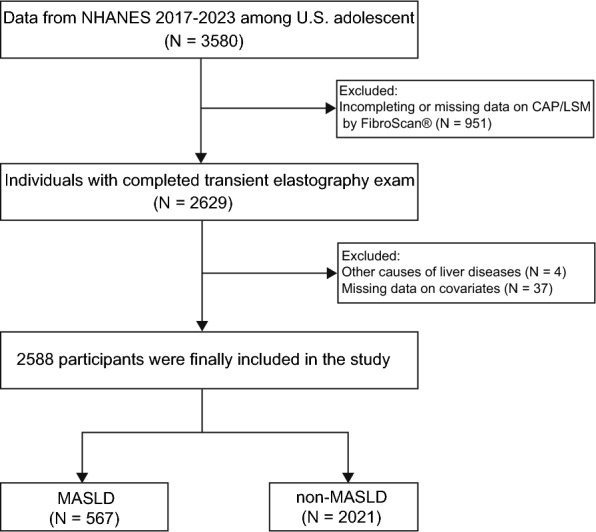
Flow chart of the study cohort selection. NHANES, National Health and Nutrition Examination Survey; MASLD, metabolic dysfunction-associated steatotic liver disease; CAP, controlled attenuation parameter; LSM, liver stiffness measurement

This study analyzed the data from using the U.S. National Health and Nutrition Examination Surveys (NHANES) covering two periods from 2017 until March 2020 (pre-pandemic data) and from August 2021 to August 2023. The NHANES represent a comprehensive, continual cross-sectional study that systematically collects a diverse array of data through interviews, physical examinations, and laboratory assessments aimed at exploring various health-related issues. The National Center for Health Statistics (NCHS) Research Ethics Review Board approved all NHANES protocols, with written informed consent (and parental assent for minors) obtained during data collection. We employed the CAP for the diagnosis of steatotic liver disease (SLD). Participant selection followed a systematic process as illustrated in Fig. 1. Our exclusion criteria included: (1) age ≥ 20 years; (2) lacking complete components for CAP or liver stiffness measurement (LSM); (3) excessive alcohol consumption (average alcohol consumption >30 g/day for males and 20 g/day for females) [5, 16]; and (4) other pre-existing liver conditions, including viral hepatitis infection (defined as a positive HCV RNA, HCV antibody or HBsAg test) and autoimmune hepatitis. Subjects with incomplete data were excluded to ensure robustness in our analysis. Consequently, a final cohort of 2588 participants were included in the study.

### Clinical and laboratory evaluations

Variables encompassing gender, age, race/ethnicity, medical history, blood pressure, waist circumference (WC), laboratory tests, and transient elastography examinations, and transient elastography examinations, which were previously known to be associated with liver steatosis or fibrosis, were sourced from NHANES data set. Age was categorized into three groups: 12 to 14 years, 15 to 17 years, and 18 to 19 years [17]. Race/ethnicity was classified into categories of non-Hispanic white, non-Hispanic black, Hispanic, non-Hispanic Asian, and other. Data on daily alcohol consumption was collected through two 24-hour recalls, with excessive alcohol consumption defined as an average intake exceeding 30 grams per day for men and 20 grams per day for women [18, 19]. In this study, the missForest algorithm was utilized to address the missing values (< 25%) in the NHANES datasets (Table S1).

### Definition of MASLD

The evaluation of liver steatosis and fibrosis was conducted using the FibroScan^®^ model 502 V2 Touch (Echosens, Paris, France) to conduct the elastography exam in the NHANES Mobile Examination Center, and the values of median CAP and LSM were calculated by the device along with the interquartile range [20, 21]. Complete data were defined as fasting time of at least 3 hours, 10 or more complete stiffness measures, and a liver stiffness interquartile range/median < 30%. In this study, we used two CAP score cut-off values, 248 dB/m and 263 dB/m, for the diagnosis of SLD [22]. MASLD is defined as SLD in addition to at least one of the five cardiometabolic criteria without viral hepatitis or significant alcohol consumption (Table S2): [5, 23] (1) overweight or obesity (body mass index ≥ 85th percentile for age/sex or waist circumference ≥ 95th percentile); (2) fasting blood glucose levels of ≥ 100 mg/dL or glycosylated hemoglobin levels of ≥ 5.7%, a history of diabetes, or currently receiving treatment for diabetes; (3) blood pressure (BP) ≥ 130/80 mmHg or ≥ 95th percentile in participants under 13 years, or BP ≥ 130/85 mmHg in those aged 13 or undergoing specific antihypertensive drug treatment; (4) plasma triglyceride (TG) ≥ 150 mg/dL in participants aged 10 and older, TG ≥ 100 mg/dL in participants younger than 10 years or currently receiving lipid-lowering therapy; (5) plasma high-density lipoprotein cholesterol (HDL-C) ≤ 40 mg/dL or the use of lipid-lowering medication. In this study, a median LSM value ≥7.4 kPa was used to identify subjects with fibrosis.

### Statistical analysis

All data descriptions and statistical analyses were complexly weighted using the "Survey" package by R software (version 4.4.1) and GraphPad Prism 10.0. In this study, the sample weights, stratification, and clustering used in the NHANES study were applied to all statistical analyses to account for the complex, multistage sampling design used to select a representative sample of the noninstitutionalized civilian U.S. population. Continuous variables were reported as mean ± standard deviation for those following a normal distribution and homogeneity of variance, while non-normally distributed data were expressed as median with interquartile range. Categorical variables were summarized as percentages and frequencies. Variance inflation factors (VIFs) were used to evaluate multicollinearity. Multivariable logistic regression models were used to assess the association between MASLD and risk factors. We applied sampling weights to account for the complex design of NHANES, thereby reconstructing representative data for the US population. Age-adjusted prevalence estimates were calculated using the direct method consistent with the 2000 US Census standard population. Logistic regression analysis was performed to evaluate the effect of demographic and metabolic variables on the risk of having MASLD and fibrosis. A p-value of less than 0.05 was considered statistically significant.

## Results

### Baseline characteristics of the subjects

This study cohort finally included a total of 2588 US adolescents in 2017 through 2023. The mean (SE) age of this cohort was age was 15.4 (2.3) years, 52.8% were male, and 47.2% were female. The racial and ethnic composition of the NHANES 2017-2023 population closely reflects that of the US, Specifically, 27.2% identified as Hispanic, 35.3% as non-Hispanic White, 20.0% as non-Hispanic Black, 8.7% as non-Hispanic Asian, and 8.9% as belonging to other racial/ethnic categories or identifying as multiracial. Participant characteristics are summarized in Table 1. Individuals with MASLD were older and had a higher representation of men and Hispanics compared to those without MASLD; this trend was similarly observed in the population with MASLD-related fibrosis. Notably, a lower proportion of MASLD was found among non-Hispanic blacks, whereas the proportion of MASLD-related fibrosis was higher in this group. Furthermore, individuals with MASLD demonstrated elevated BMI, WC, total cholesterol levels, and HbA1c levels, while exhibiting lower HDL-C levels (all *P* < 0.001). Additionally, individuals with MASLD and MASLD-related fibrosis displayed higher proportions of CAP and LSM compared to those without MASLD and MASLD-related fibrosis.

**Table 1:** Baseline characteristic of the study population

	Overall (N = 2588)	No MASLD (N = 2021)	MASLD (N = 567)	*P*-value	No fibrosis in MASLD (N = 566)	Fibrosis in MASLD (N = 61)	*P*-value
Gender (n, %)				0.034			0.814
Male	1366 (52.8)	1044 (51.7)	322 (56.8)		286 (56.5)	36 (59.0)	
Female	1222 (47.2)	977 (48.3)	245 (43.2)		220 (43.5)	25 (41.0)	
Age (years)	15.4 (2.3)	15.4 (2.3)	15.6 (2.2)	0.053	15.5 (2.2)	16.4 (2.1)	0.003
Race (n, %)				<0.001			0.658
Non-Hispanic White	913 (35.3)	734 (36.3)	179 (31.6)		164 (32.4)	15 (24.6)	
Non-Hispanic Black	517 (20.0)	422 (20.9)	95 (16.8)		82 (16.2)	13 (21.3)	
Hispanic	704 (27.2)	498 (24.6)	206 (36.3)		182 (36.0)	24 (39.3)	
Non-Hispanic Asian	224 (8.7)	187 (9.3)	37 (6.5)		34 (6.7)	3 (4.9)	
Other Race	230 (8.9)	180 (8.9)	50 (8.8)		44 (8.7)	6 (9.8)	
SBP (mmHg)	108.5 (10.3)	108.2 (10.1)	109.7 (10. 9)	0.001	109.4 (10.9)	112.7 (10.8)	0.022
DBP (mmHg)	64.7 (8.2)	63.7 (7.7)	68.4 (8.9)	<0.001	67.8 (8.6)	73.7 (9.0)	<0.001
HDL (mg/dl)	51.9 (10.7)	53.6 (10.5)	45.6 (8.9)	<0.001	45.9 (9.0)	43.2 (7.2)	0.023
TC (mg/dl)	153.8 (27.3)	152.0 (26.8)	160.2 (28.1)	<0.001	160.2 (27.8)	160.4 (30.8)	0.968
HbA1c (%, mmol/mol, mean (SD))	5.2 (0.3)	5.229 (0.3)	5.3 (0.3)	<0.001	5.3 (0.3)	5.4 (0.3)	0.019
BMI (kg/m^2^)	24.6 (6.5)	22.5 (4.5)	32.1 (7.0)	<0.001	31.3 (6.3)	38.3 (9.2)	<0.001
BMI Category (n, %)				<0.001			0.001
Underweight	103 (4.03)	102 (5.12)	1 (0.18)		1 (0.20)	0 (0.00)	
Normalweight	1409 (55.1)	1367 (68.6)	42 (7.4)		40 (7.9)	2 (3.3)	
Overweight	440 (17.2)	313 (15.7)	127 (22.5)		124 (24.6)	3 (4.9)	
Obese	605 (23.7)	210 (10.5)	395 (69.9)		339 (67.3)	56 (91.8)	
WC (cm)	82.8 (15.7)	77.5 (11.0)	101.8 (15.4)	<0.001	100.1 (14.1)	115. 8 (18.3)	<0.001
LSM	5.02 (2.34)	4.84 (1.77)	5.68 (3.65)	<0.001	4.99 (1.03)	11.42 (8.90)	<0.001
CAP	219.96 (53.71)	199.22 (36.72)	293.86 (36.98)	<0.001	290.64 (34.57)	320.57 (45.111)	<0.001
Year				0.483			0.161
2017-2020	1599 (61.8)	1241 (61.4)	358 (63.1)		314 (62.1)	44 (72.1)	
2021-2023	989 (38.2)	780 (38.6)	209 (36.9)		192 (37.9)	17 (27.9)	

Continuous variables are presented as mean ± SD and (min–max), and categorical data as n (%). MASLD, metabolic dysfunction-associated steatotic liver disease; SBP, systolic blood pressure; DBP, diastolic blood pressure; HDL, high‐density lipoprotein; TC, total cholesterol; WC, waist circumference; CAP, controlled attenuation parameter; LSM, liver stiffness measurement.

### Prevalence of MASLD and MASLD-related fibrosis among adolescent

**Fig. 2:**
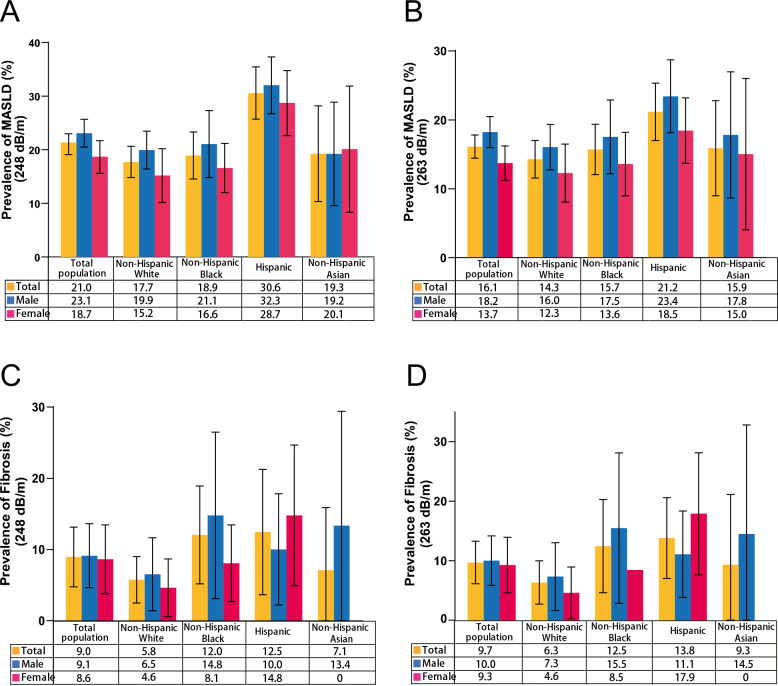
Age-adjusted prevalence of MASLD and fibrosis subcategories by sex and race/ethnicity in the United States adolescent (ages 12–19), 2017–2023. **A** Age-adjusted prevalence of MASLD defined as CAP score ≥ 248 dB/m. **B** Age-adjusted prevalence of MASLD defined as CAP score ≥ 263 dB/m. **C** Age-adjusted prevalence of MASLD-related fibrosis defined as CAP score ≥ 248 dB/m. **D** Age-adjusted prevalence of MASLD-related fibrosis defined as CAP score ≥ 263 dB/m. MASLD, metabolic dysfunction-associated steatotic liver disease; CAP, controlled attenuation parameter

The weighted age-adjusted prevalence of MASLD (248 dB/m) was 21.0% (95% confidence interval [CI] 19.1–23.0%), and MASLD-related fibrosis was 9.0% (95% CI 4.8–13.2%). As expected, the prevalence of MASLD decreased to 16.1% (95% CI 14.4–17.8) in a sensitivity analysis where the CAP threshold for SLD was adjusted to ≥ 263 dB/m. As shown in Fig. 2A, the prevalence of MASLD was notably higher in Hispanics (30.6%, 95% CI 25.7–35.46), followed by non-Hispanic Asians (19.3%, 95% CI 10.3–28.2), non-Hispanic blacks (18.9%, 95% CI 14.6–23.3) and non-Hispanic whites (17.7%, 95% CI 14.8–20.6). In terms of MASLD-related fibrosis, the prevalence was substantially higher in Hispanics (12.5%, 95% CI 3.7–21.3), followed by non-Hispanic blacks (12.0%, 95% CI 5.2–18.9), non-Hispanic Asians (7.1%, 95% CI −1.7–15.89) and non-Hispanic whites (5.8%, 95% CI 2.5–9.0) as shown in Fig. 2C. The prevalence was comparable across various races/ethnicities and sexes when comparing results based on a cut-off of 263 dB/m (Fig. 2B, D). The prevalence of MASLD and MASLD-related fibrosis is predominantly higher in men than in women; however, among non-Hispanic Asians, the prevalence of MASLD shows a slight increase in females (248 dB/m).

### Prevalence of MASLD and MASLD-related fibrosis categorized by weight and diabetes

**Fig. 3:**
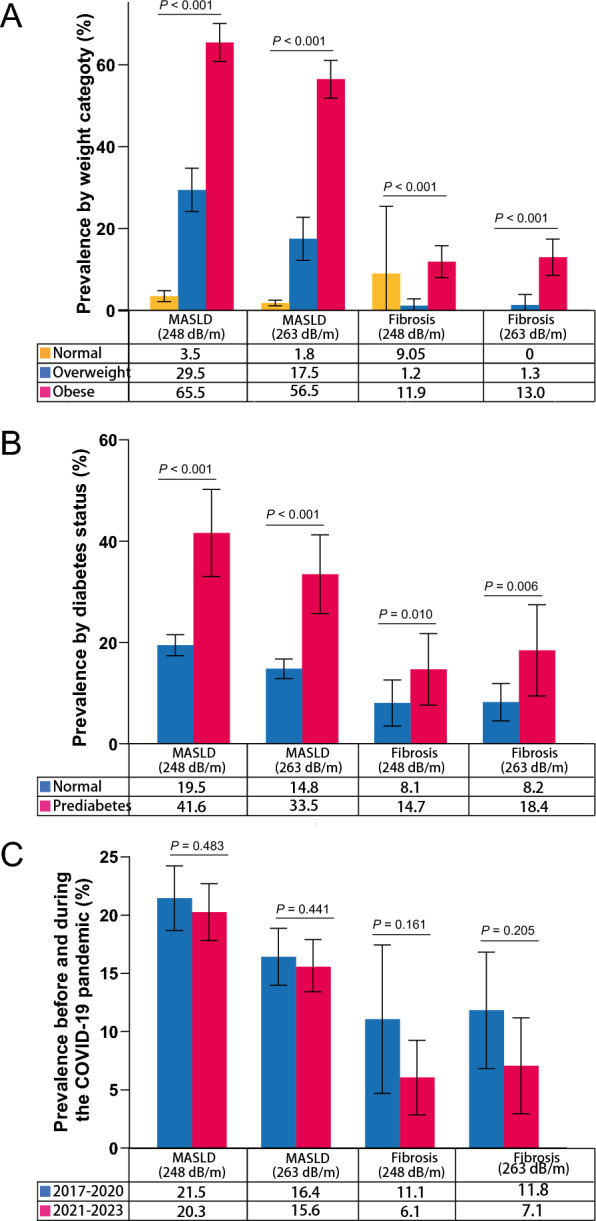
Age-adjusted prevalence of fibrosis and cirrhosis among individuals with MASLD and fibrosis by weight, diabetes, and comparison in the United States adolescent, 2017–2023. **A** Age-adjusted prevalence of MASLD and MASLD-related fibrosis subcategories by weight. **B** Age-adjusted prevalence of MASLD and MASLD-related fibrosis subcategories by diabetes status. **C** Comparison between the pre-COVID-19 era (2017–2020) and the COVID-19 era (2021–2023) regarding the prevalence of MASLD and fibrosis. MASLD, metabolic dysfunction-associated steatotic liver disease

When stratified by weight, the weighted prevalence of MASLD differed across the various weight categories (Fig. 3A). Among normal weight adolescents, the prevalence was 3.5% (95% CI 2.2–4.8), with 9.1% exhibiting MASLD-related fibrosis. In overweight adolescents, the prevalence increased to 29.5% (95% CI 24.2–34.7), with a corresponding fibrosis rate of 1.2%. The highest prevalence was observed in obese adolescents, where 65.5% (95% CI 60.8–70.1) demonstrated MASLD, and 11.9% had MASLD-related fibrosis, based on a cutoff of 248 dB/m. When a cutoff value of 263 dB/m was applied to optimize both specificity and sensitivity, the weighted overall prevalence of MASLD was 1.8% (95% CI: 1.1–2.5), 17.5% (95% CI: 12.2–22.8), and 56.5% (95% CI: 51.8–61.1) among individuals with normal weight, overweight, and obesity, respectively. The corresponding prevalence of MASLD-related fibrosis was 0%, 1.3% (95% CI: −1.2–3.9), and 13.0% (95% CI: 8.6–17.4), respectively.

The weighted prevalence of MASLD and MASLD-related fibrosis was found to be significantly associated with diabetes status. As depicted in Fig. 3B, the age-adjusted prevalence of MASLD (248 dB/m) was significantly higher in individuals with prediabetes (41.6%, 95% CI 33.1–50.2%) compared to those with normal glucose tolerance (19.5%, 95% CI 17.4–21.5%). When employing an alternate cut-off of ≥ 263 dB/m, the prevalence of MASLD in individuals with normal glucose tolerance was 14.8%, while in those with prediabetes, it increased to 33.5%. Among individuals diagnosed with MASLD, the age-adjusted prevalence of fibrosis exhibited a similar trend, being more pronounced in individuals with prediabetes compared to those with normal glucose tolerance.

### Comparison of prevalence of MASLD and MASLD-related fibrosis between the pre-COVID-19 era and the COVID-19 era

Comparison between the two NHANES cycles (2017 to March 2020 vs. August 2021 to August 2023) showed similar demographic and clinical characteristics, except for race/ethnicity (Table S3).

As shown in Fig. 3C, the prevalence of MASLD showed a slight decrease during the COVID-19 era, employing cut-off values of ≥ 248 dB/m and 263 dB/m, although this reduction was not statistically significant. We observed an obvious decrease in the prevalence of MASLD-related fibrosis during the COVID-19 era compared to the pre-COVID-19 era. Nevertheless, there remained no significant difference in the proportion of MASLD-related fibrosis between the two time periods.

### Predictor for MASLD and MASLD-related fibrosis among adolescent

Table 2 presents the risk factors associated with MASLD using CAP thresholds of ≥ 248 dB/m and 263 dB/m. In multivariable logistic regression, male sex, non-Hispanic Asian ethnicity, waist circumference, overweight, and obesity were consistently identified as independent risk factors across both cutoffs. In terms of MASLD-fibrosis, an increased waist circumference is associated with a heightened risk of fibrosis in individuals diagnosed with MASLD (Table S4). Additionally, the VIFs for all variables were below 2.5, indicating no evidence of multicollinearity. Meanwhile, the models also demonstrated excellent discrimination, with an area under the curve greater than 0.90. Model fit, summarized by Nagelkerke’s pseudo-R^2^, had values of 0.381 for 248 dB/m and 0.340 for 263 dB/m model, respectively, indicating a meaningful proportion of explained outcome variation (Figures S1 and S2).

**Table 2:** Multivariable odds ratio of predictors for MASLD

**Variables**	MASLD (248 dB/m) OR (95% CI)	*P*-value	MASLD (263 dB/m) OR (95% CI)	*P*-value
Gender (n, %)				
Female	1		1	
Male	1.32 (1.01–1.73.01.73)	0.045	1.47 (1.09–1.96.09.96)	0.010
Age (years)	0.94 (0.88–1.00.88.00)	0.065	0.97 (0.90–1.04.90.04)	0.333
Race (n, %)				
Non-Hispanic White	1		1	
Non-Hispanic Black	0.69 (0.46–1.04.46.04)	0.074	0.68 (0.44–1.04.44.04)	0.074
Hispanic	1.91 (1.38–2.64.38.64)	<0.001	1.39 (0.99–1.97.99.97)	0.060
Non-Hispanic Asian	1.96 (1.13–3.38.13.38)	0.016	2.00 (1.11–3.59.11.59)	0.021
Other Race	0.94 (0.58–1.53.58.53)	0.811	0.86 (0.51–1.44.51.44)	0.560
HDL (mg/dl)	0.98 (0.97–1.00.97.00)	0.018	0.99 (0.97–1.00.97.00)	0.125
TC (mg/dl)	1.00 (1.00–1.01.00.01)	0.075	1.01 (1.00–1.01.00.01)	0.002
HbA1c (%, mmol/mol)	1.50 (0.86–2.62.86.62)	0.155	1.48 (0.82–2.68.82.68)	0.193
WC (cm)	1.09 (1.07–1.11.07.11)	<0.001	1.09 (1.07–1.11.07.11)	<0.001
BMI Category (n, %)				
Under/normal weight	1		1	
Overweight	4.60 (3.00–7.05.00.05)	<0.001	4.51 (2.69–7.57.69.57)	<0.001
Obese	5.26 (2.99–9.24.99.24)	<0.001	6.69 (3.56–12.56.56.56)	<0.001
Diabetes status (n, %)				
Normal	1		1	
Pre-/diabetes	0.94 (0.57–1.56.57.56)	0.822	0.98 (0.59–1.63.59.63)	0.944

Continuous variables are presented as mean ± SD and (min–max), and categorical data as n (%). MASLD, metabolic dysfunction-associated steatotic liver disease; OR, odds ratio; HDL, high‐density lipoprotein; TC, total cholesterol; WC, waist circumference, HbA1c, Hemoglobin A1C.

## Discussion

In this large population-based study, we analyzed a representative sample of American adolescents from NHANES 2017 to 2023. The prevalence of MASLD was 21.0%, and 9.0% had MASLD-related fibrosis based on a CAP threshold of ≥ 248 dB/m. Notable disparities in prevalence by race and ethnicity were observed, with a higher prevalence of MASLD among Hispanic and non-Hispanic Asian adolescents, and a greater prevalence of fibrosis among Hispanic and non-Hispanic Black adolescents. Although no significant differences in the prevalence of MASLD and fibrosis were found between the pre-COVID-19 era (2017–2020) and the COVID-19 period (2021–2023), overweight, obesity, and prediabetes were strongly associated with an increased risk of MASLD and related fibrosis in adolescents.

Our results reveal a slightly lower prevalence of MASLD compared to comparable data on adolescents aged 12 to 19 from the 2017 to 2020 NHANES cycles, which reported a prevalence of MASLD, defined as CAP values of ≥ 248 dB/m, at 23.77% [9]. This observed gap may be attributed to the stringent inclusion criteria employed in our study, which resulted in the exclusion of individuals with autoimmune hepatitis and those exceeding the specified thresholds for severe alcohol consumption (≥ 30 g/day for males and ≥ 20 g/week for females). However, when MASLD was defined using a CAP threshold of ≥ 263 dB/m, our findings were consistent with previous data reporting a prevalence of 16.8% among US adolescents aged 12 to 17 years [24]. Our analysis provides a more accurate representation than aforementioned studies, as it incorporates recent data from 2017 to 2023, employs a more conservative cutoff value for the CAP score (≥ 248 and ≥ 263 dB/m), and utilizes the newly established definition of MASLD. Consistent with previous studies, we observed a higher prevalence of MASLD in males and a similar trend by race and ethnicity, with the highest prevalence in Hispanics, followed by non-Hispanic Black and non-Hispanic Asian adolescents [9, 24].

The COVID-19 pandemic has significantly influenced the prevalence of MASLD, largely due to lifestyle changes during nationwide lockdowns. Many individuals experienced decreased physical activity levels and increased sedentary behavior during this period, compounded by a rise in unhealthy dietary habits and subsequent weight gain. Evidence suggests that these lifestyle alterations have the potential to adversely impact MASLD prevalence, as sedentary behavior is associated with the development of metabolic dysfunction and hepatic conditions [25]. Previous studies reported a higher proportion of fibrosis among US adults with MASLD during the COVID-19 period compared to the pre-COVID-19 period, although the differences were not statistically significant [26, 27]. Pandemic-induced lifestyle alterations, including increased indoor time, reduced physical activity, and heightened alcohol consumption, all of which have adversely affected liver health, particularly among individuals predisposed to SLD. Patients with metabolic dysfunction were often deprived of essential monitoring and therapeutic interventions during this period, further contributing to fibrosis progression related to SLD, as well as increased all-cause and HCC-related mortality [25, 28, 29]. However, in this study, we did not observe an increase in the prevalence of fibrosis among adolescents. This finding could be attributed to a reduction in routine health screenings, leading to delayed diagnoses of cases. Furthermore, our study's focus on adolescents is pertinent, as the development of fibrosis can take several decades, making early identification challenging. A mean follow-up of 10.3 years among 51 participants revealed that one-third of young adults with childhood obesity developed non-alcoholic fatty liver disease (NAFLD), while one-third experienced resolution of steatosis. At follow-up, 6% of individuals with NAFLD had progressed to advanced fibrosis [30].

The prevalence of MASLD across ethnic groups have reported significant differences in both adults and children [26, 31, 31–33]. Consistent with previous findings, our study also observed significant variation in MASLD prevalence among adolescents of different racial and ethnic backgrounds. Specifically, Hispanic and non-Hispanic Asian adolescents tend to present with dyslipidemia and exhibited a higher prevalence of MASLD, while non-Hispanic Black adolescents are more likely to exhibit impaired glucose metabolism and appeared relatively protected against hepatic fat accumulation. Several factors may account for these ethnic disparities. Firstly, genetic variation, particularly in the PNPLA3 gene, has been shown to contribute to differences in hepatic fat content and susceptibility to NAFLD [34–36]. Tricò et al. demonstrated that incorporating the three major single nucleotide polymorphisms (SNPs) associated with NAFLD, including rs738409 in the PNPLA3 gene, rs1260326 in the GCKR gene, and rs58542926 in the TM6SF2 gene, significantly enhances the predictive capacity for changes in hepatic fat fraction during follow-up assessments [37]. Particularly, the minor allele frequencies of rs738409 and rs1260326 are highest among Hispanics and lowest among individuals of African descent. Conversely, the rs6006460[T] variant in PNPLA3, which encodes the S453I substitution, is linked to reduced hepatic fat content in African Americans, potentially explaining their lower risk of SLD despite similar rates of obesity and insulin resistance. Secondly, sociodemographic factors may further contribute to underdiagnosis among certain groups [38]. Non-Hispanic Asians and Blacks in the US often represent underserved populations with limited access to routine health screening, potentially leading to underestimation of MASLD prevalence. Thirdly, racial differences in fat distribution may play a role. Visceral adipose tissue, the primary source of free fatty acids for hepatic triglyceride synthesis, tends to be less abundant in Blacks compared with Whites and Hispanics [39]. This physiologic variation likely contributes to the lower propensity for MASLD observed in non-Hispanic Black adolescents, further highlighting the ethnic heterogeneity in disease prevalence.

Currently, various diagnostic modalities for MASLD are available; however, no single optimal tool has been established [40]. Studies have shown that the fatty liver index (FLI), hepatic steatosis index (HSI), and U.S. fatty liver index (USFLI) are significantly associated with pediatric MASLD, even after adjusting for age, sex, obesity, and diabetes [41, 42]. Moreover, FLI and HSI exhibit excellent predictive accuracy for diagnosing MASLD across different cohorts and subgroups, as well as for distinguishing patients with severe MASLD from those with normal alanine aminotransferase (ALT) levels [18, 40, 43]. Although ALT is recommended in clinical guidelines as a cost-effective and accessible screening tool for pediatric NAFLD, its utility is limited by low sensitivity and its inability to directly reflect underlying metabolic dysfunction [44, 45]. A study using NHANES data from 2017 to 2020 reported a suspected MASLD prevalence of 11.3% among adolescents aged 12 to 19 years based on elevated ALT, which is notably lower than our observed prevalence of 21.0% [46]. In comparison, Ma et al. reported a prevalence of 25.1% using a CAP threshold of ≥ 240 dB/m, while Zheng et al. reported a prevalence of 23.77% using a CAP threshold of ≥ 248 dB/m, both based on US adolescents aged 12 to 19 years using the same NHANES cycle [9, 47].

We further analyzed risk factors associated with MASLD. In multivariable logistic regression, male sex, non-Hispanic Asian ethnicity, increased waist circumference, overweight, and obesity were independently associated with a higher risk of MASLD. Waist circumference was also independently associated with an elevated risk of MASLD-related fibrosis. In addition, the highest prevalence of MASLD was observed among adolescents with prediabetes, who also showed a greater extent of fibrosis. Beyond the risk of progression from simple steatosis to metabolic dysfunction-associated steatohepatitis (MASH), fibrosis, and cirrhosis, adolescents and children with MASLD have been shown to face significantly higher all-cause mortality and mortality from liver disease, cardiometabolic conditions, and cancer [2, 9, 18]. One nationwide, cohort study children and young adults (≤ 25 years) with biopsy-confirmed MASLD reported an absolute all-cause mortality risk of 7.7% among MASLD patients, compared to 1.1% in controls [48]. These findings highlight the urgent need for strategies to reduce the disease burden and long-term consequences of MASLD in adolescents.

This study has several strengths. We leveraged the latest available cycles of NHANES from 2017 to 2023, providing nationally representative estimates across both pre- COVID-19 and COVID-19 periods. The use of transient elastography allowed for more accurate estimation of MASLD prevalence across time periods and among subgroups stratified by sex, race and ethnicity, weight status, and diabetes. We applied CAP thresholds of 248 dB/m and 263 dB/m, which are supported by prior literature and may be more appropriate for adolescents than the 285 dB/m threshold used in some studies that may underestimate MASLD prevalence. In addition, we performed comprehensive logistic regression analyses to identify predictors of MASLD and MASLD-related fibrosis.

Nevertheless, our study is subject to several limitations. Firstly, despite the employment of recognized CAP score cutoffs, the absence of universally accepted guidelines for both CAP scores and liver stiffness measurements is a significant drawback [9, 24, 49]. Secondly, proposed LSM cut-offs vary, and no universal threshold has been established in adolescents. We defined fibrosis using an LSM ≥ 7.4 kPa but were unable to further classify fibrosis stages (e.g., significant fibrosis or cirrhosis). Nobili et al. reported high sensitivity (100%) and specificity (92%) for pediatric fibrosis at 7.4 kPa [50]. Ma et al. estimated a fibrosis prevalence of 8.9% (95% CI 3.7–14.0%) in adolescents with MASLD using the same threshold [47], similar to our estimate of 9.0% (95% CI 4.8–13.2%). Although these findings support the robustness of our results, future longitudinal studies should validate them, and sensitivity analyses using alternative LSM thresholds (e.g., 6.5 and 8.0 kPa) are warranted. Additionally, the operator-dependent nature of CAP measurements and variability in cutoff values may result in inaccuracies, potentially leading to the overestimation or underestimation of MASLD prevalence. Meanwhile, the missingness of certain variables may not be completely at random and could introduce bias. Fourthly, the post-pandemic NHANES data (2021-2023) are an interim, partially released dataset and are not fully harmonized with pre-pandemic cycles due to changes in sampling methodology. Consequently, comparisons between pre- and post-pandemic periods should be interpreted with caution and are considered descriptive in nature. Finally, the absence of liver biopsy for histological validation, considered the gold standard for diagnosis, must be recognized as a limitation of this study.

In conclusion, this cross-sectional, nationally representative analysis shows that approximately one-fifth affected by MASLD and one in ten exhibiting MASLD-related fibrosis. Significant differences were observed by sex and race and ethnicity, with a higher prevalence in males, and Hispanic adolescents showing the highest prevalence of both MASLD and fibrosis. These findings highlight populations that may benefit from increased clinical vigilance and identify priority groups for future longitudinal studies to determine the natural history of MASLD in adolescents. Furthermore, early detection and prompt intervention should be prioritized to prevent progression to fibrosis and other complications.

## Supplementary Information

Additional file 1.

## Data Availability

The datasets supporting the conclusions of this article was available in the public repository as described below. The authors do not own the data. National Health and Nutrition Examination Survey data are available at (http:/www.cdc.gov/nchs/nhanes).
