# Supplementary material for: Prevalence of MASLD and fibrosis assessed by transient elastography in U.S. adolescents: insights from NHANES 2017-2023
Source: Diabetol Metab Syndr. 2025 Dec 30;17:462. doi: 10.1186/s13098-025-02024-9 (PMC12751582; doi:10.1186/s13098-025-02024-9)
Supplement: Supplementary file 1 — Additional file 1. [file 13098_2025_2024_MOESM1_ESM.pdf]

Supplementary

Prevalence of MASLD and Fibrosis Assessed by Transient Elastography in U.S. Adolescents Before and During the COVID-19 Pandemic: Insights from NHANES 2017-2023

目录

Figure S1 2
Figure S2 3
Table S1 4
Table S2 5
Table S3 6
Table S4 8

Figure S1 Variance inflation factor graph. (A) CAP ≥248 dB/m; (B) CAP ≥263 dB/m.

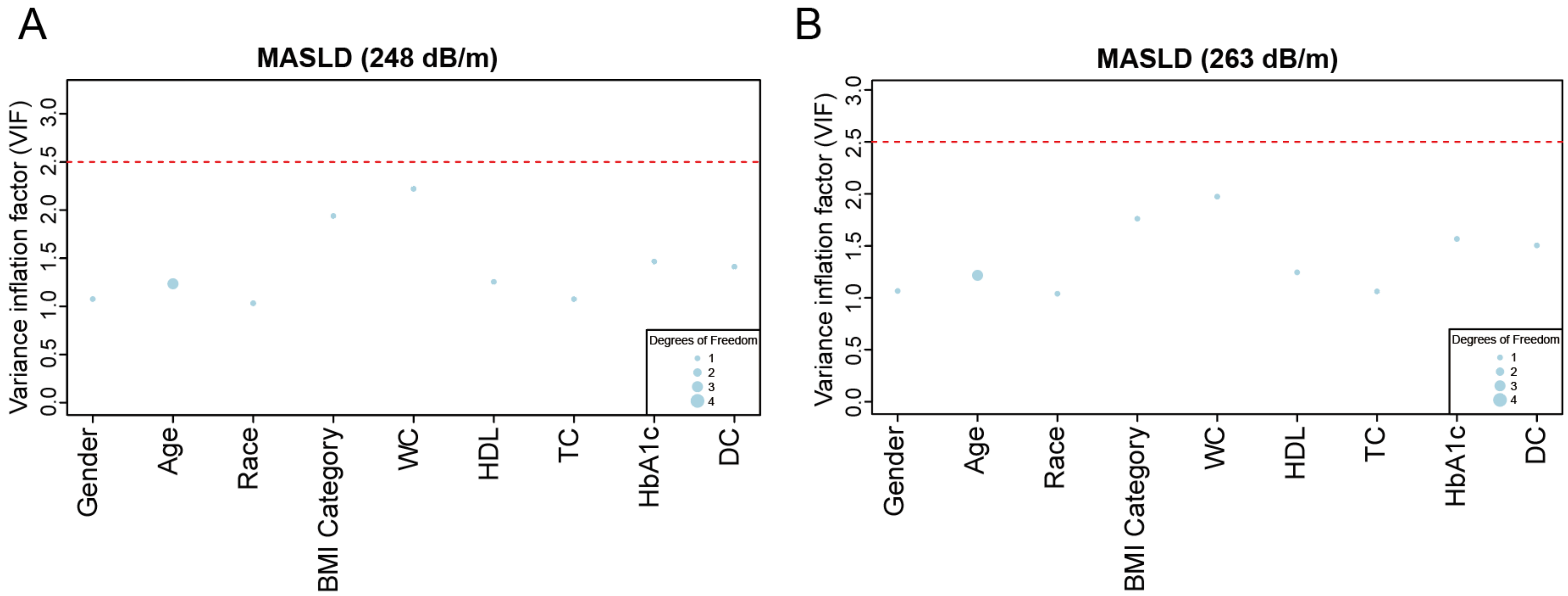

MASLD, metabolic dysfunction-associated steatotic liver disease; CAP, controlled attenuation parameter; HDL, high-density lipoprotein; TC, total cholesterol; WC, waist circumference; DC, diabetes category.

Figure S2 ROC curves and model fit for MASLD prediction models. (A) CAP ≥248 dB/m; (B) CAP ≥263 dB/m.

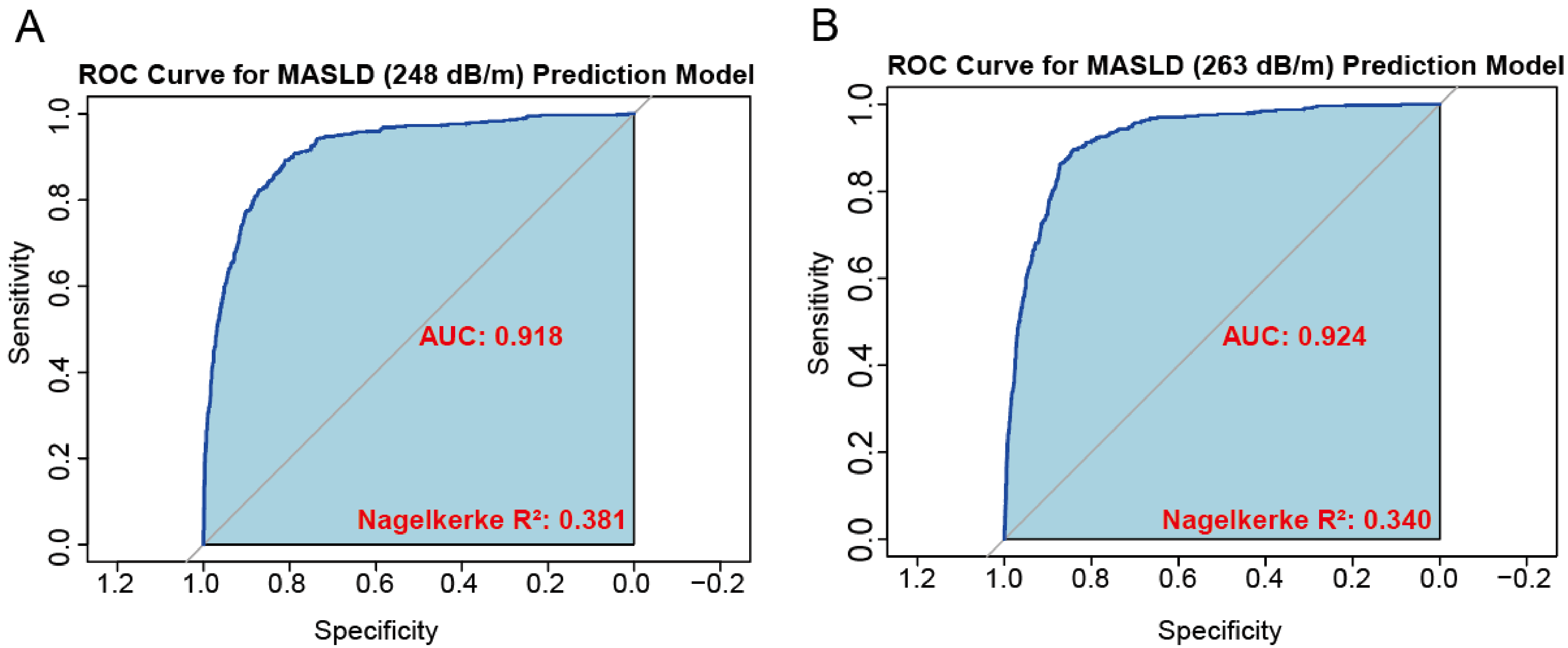

MASLD, metabolic dysfunction-associated steatotic liver disease; CAP, controlled attenuation parameter; ROC, Receiver operating characteristic, AUC, area under the curve.

Table S1 Summary of variables with missing data (<25%) in the NHANES datasets.

Variable	NHANES Dataset
	Missing Data (%)
HDL (mg/dl)	386 (14.92)
TC (mg/dl)	386 (14.92)
HbA1c (%, mmol/mol)	303 (11.72)
SBP (mmHg)	183 (7.07)
DBP (mmHg)	183 (7.07)
Waist circumference (cm)	57 (2.2)
BMI Category	35 (1.35)
BMI (kg/m^2)	19 (0.73)

HDL, high-density lipoprotein; TC, total cholesterol; WC, waist circumference, HbA1c, Hemoglobin A1C, SBP, systolic blood pressure; DBP, diastolic blood pressure

Table S2 Definition of metabolic dysfunction-associated steatotic liver disease for adolescent.

Variable	Operationalization
SLD	CAP score ≥ 248 dB/m or 263 dB/m
Overweight or obesity	body mass index ≥ 85th % for age/sex or waist circumference ≥ 95th %
Prediabetes/diabetes mellitus	fasting blood glucose levels of ≥ 100 mg/dL or glycosylated hemoglobin levels of ≥ 5.7%, a history of diabetes, or currently receiving treatment for diabetes
Raised BP	BP ≥ 130/80 mmHg or ≥ 95th percentile in participants under 13 years, or BP ≥ 130/85 mmHg in those aged 13 or undergoing specific antihypertensive drug treatment
Dyslipidemia	TG ≥ 150 mg/dL in participants aged 10 and older, TG ≥ 100 mg/dL in participants younger than 10 years or currently receiving lipid-lowering therapy
Low HDL	HDL-C ≤ 40 mg/dL or the use of lipid-lowering medication
MASLD	SLD + one or more of cardiometabolic risk factors

SLD, steatotic liver disease; BP, blood pleasure; TG, plasma triglyceride; HDL-C, high-density lipoprotein cholesterol; MASLD, metabolic dysfunction associated steatotic liver disease; CMRF, cardiometabolic risk factor.

Table S3 Characteristics of study populations based on the NHANES cycles (2017–2020 vs. 2021–2023).

	2017-2020 (N = 1599)	2021-2023 (N = 989)	*P*-value
Gender (n, %)			0.441
Male	854 (53.4)	512 (51.8)	
Female	745 (46.6)	477 (48.2)	
Age (years)	15.4 (2.2)	15.5 (2.3)	0.672
Race (n, %)			<0.001
Non-Hispanic White	523 (32.7)	390 (39.4)	
Non-Hispanic Black	385 (24.1)	132 (13.4)	
Hispanic	394 (24.6)	310 (31.3)	
Non-Hispanic Asian	156 (9.8)	68 (6.9)	
Other Race	141 (8.8)	89 (9.0)	
SBP (mmHg)	108.7 (10.1)	108.2 (10.7)	0.327
DBP (mmHg)	64.7 (8.0)	64.8 (8.6)	0.897
HDL (mg/dl)	53.6 (10.5)	45.6 (8.9)	<0.001
TC (mg/dl)	152.0 (26.8)	160.2 (28.1)	<0.001
HbA1c (mean (SD))	5.2 (0.3)	5.3 (0.3)	<0.001
BMI (kg/m^2)	24.7 (6.6)	24.4 (6.4)	0.311
BMI_Category (n, %)			0.317
Underweight	55 (3.5)	48 (4.9)	
Normalweight	868 (55.0)	541 (55.3)	
Overweight	277 (17.5)	163 (16.7)	
Obese	379 (24.0)	226 (23.1)	

WC (cm)	83.0 (16.0)	82.5 (15.4)	0.499
LSM	5.1 (2.4)	4.9 (2.2)	0.153
CAP	221.2 (53.5)	218.0 (54.0)	0.147
MASLD	83.0 (16.0)	82.5 (15.4)	0.483
No	1241 (77.6)	780 (78.9)	
Yes	358 (22.4)	209 (21.1)	

Continuous variables are presented as mean ± SD and (min–max), and categorical data as n (%). MASLD, metabolic dysfunction-associated steatotic liver disease; SBP, systolic blood pressure; DBP, diastolic blood pressure; HDL, high-density lipoprotein; TC, total cholesterol; WC, waist circumference; CAP, controlled attenuation parameter; LSM, liver stiffness measurement.

Table S4 Multivariable odds ratio of predictors for MASLD-related fibrosis.

	MASLD-related fibrosis (248 dB/m) OR (95% CI)	*P*-value	MASLD-related fibrosis (263 dB/m) OR (95% CI)	*P*-value
**CAP ≥ 248**				
Gender (n, %)				
Female	1		1	
Male	1.03 (0.56-1.89)	0. 925	1.09 (0.57-2.11)	0.792
Age (years)	1.09 (0.94-1.27)	0.260	1.09 (0.92-1.28)	0.327
Race (n, %)				
Non-Hispanic White	1		1	
Non-Hispanic Black	1.03 (0.40-2.66)	0.946	0.83 (0.30-2.32)	0.721
Hispanic	1.80 (0.86-3.78)	0.121	1.87 (0.83-4.17)	0.128
Non-Hispanic Asian	1.70 (0.42-6.95)	0.459	1.84 (0.42-8.03)	0.418
Other Race	1.30 (0.42-4.05)	0.645	1.48 (0.45-4.85)	0.514
HDL (mg/dl)	1.00 (0.96-1.04)	0.875	1.00 (0.95-1.04)	0.885
TC (mg/dl)	1.00 (0.99-1.01)	0.439	1.00 (0.99-1.01)	0.582
HbA1c (mean (SD))	1.54 (0.49-4.80)	0.459	2.01 (0.58-6.95)	0.271
WC (cm)	1.06 (1.03-1.09	<0.001	1.06 (1.03-1.09)	<0.001
BMI_Category (n, %)				
Under/normal weight	1		1	
Overweight	0.30 (0.05-1.91)	0.200	590805.98 (0.00-Inf)	0.986
Obese	0.54 (0.10-2.93)	0.479	1269126.34 (0.00-Inf)	0.985
Diabetes status (n, %)				
Normal	1		1	

Pre-/diabetes	1.14 (0.51-2.55)	0.755	1.18 (0.50-2.80)	0.710

Continuous variables are presented as mean ± SD and (min–max), and categorical data as n (%). MASLD, metabolic dysfunction-associated steatotic liver disease; OR, odds ratio; HDL, high-density lipoprotein; TC, total cholesterol; WC, waist circumference, HbA1c, Hemoglobin A1C.
